# Genomic profiles of IDH-mutant gliomas: *MYCN-*amplified IDH-mutant astrocytoma had the worst prognosis

**DOI:** 10.1038/s41598-023-32153-y

**Published:** 2023-04-25

**Authors:** Kwanghoon Lee, Seong-Ik Kim, Eric Eunshik Kim, Yu-Mi Shim, Jae-Kyung Won, Chul-Kee Park, Seung Hong Choi, Hongseok Yun, Hyunju Lee, Sung-Hye Park

**Affiliations:** 1 Department of Pathology, College of Medicine, Seoul National University Hospital, Seoul, Republic of Korea; 2 Department of Neurosurgery, College of Medicine, Seoul National University Hospital, Seoul, Republic of Korea; 3 Department of Radiology, College of Medicine, Seoul National University Hospital, Seoul, Republic of Korea; 4 Department of Genomic Medicine, College of Medicine, Seoul National University Hospital, Seoul, Republic of Korea; 5 School of Electrical Engineering and Computer Science, Gwangju Institute of Science and Technology, Gwangju, 61005 Republic of Korea; 6 Artificial Intelligence Graduate School, Gwangju Institute of Science and Technology, Gwangju, 61005 Republic of Korea; 7 Neuroscience Research Institute, Seoul National University College of Medicine, Seoul, Republic of Korea

**Keywords:** Cancer, Genetics, Molecular biology, Medical research, Oncology

## Abstract

This study aimed to find any ambiguous genetic outlier for “oligodendroglioma, IDH-mutant and 1p/19q-codeleted (O_IDH_mut)” and “astrocytoma, IDH-mutant (A_IDH_mut)” and to redefine the genetic landscape and prognostic factors of IDH-mutant gliomas. Next-generation sequencing (NGS) using a brain tumor-targeted gene panel, methylation profiles, and clinicopathological features were analyzed for O_IDH_mut (n = 74) in 70 patients and for A_IDH_mut (n = 95) in 90 patients. 97.3% of O_IDH_mut and 98.9% of A_IDH_mut displayed a classic genomic landscape. Combined *CIC* (75.7%) and/or *FUBP1* (45.9%) mutations were detected in 93.2% and *MGMTp* methylation in 95.9% of O_IDH_mut patients. In A_IDH_mut, *TP53* mutations were found in 86.3% and combined *ATRX* (82.1%) and *TERTp* (6.3%) mutations in 88.4%. Although there were 3 confusing cases, NOS (not otherwise specified) category, based on genetic profiles, but they were clearly classified by combining histopathology and DKFZ methylation classifier algorithms. The patients with *MYCN* amplification and/or *CDKN2A/2B* homozygous deletion in the A_IDH_mut category had a worse prognosis than those without these gene alterations and *MYCN*-amplified A_IDH_mut showed the worst prognosis. However, there was no prognostic genetic marker in O_IDH_mut. In histopathologically or genetically ambiguous cases, methylation profiles can be used as an objective tool to avoid a diagnosis of NOS or NEC (not elsewhere classified), as well as for tumor classification. The authors have not encountered a case of true mixed oligoastrocytoma using an integrated diagnosis of histopathological, genetic and methylation profiles. *MYCN* amplification, in addition to *CDKN2A/2B* homozygous deletion, should be included in the genetic criteria for CNS WHO grade 4 A_IDH_mut.

## Introduction

Astrocytoma, isocitrate dehydrogenase (IDH)-mutant (A_IDH_mut) and oligodendroglioma, IDH-mutant and 1p/19q-codeleted (O_IDH_mut) are the 2nd and 3rd most common diffuse infiltrating gliomas in adults after glioblastoma, IDH-wildtype (GBM). According to a meta-analysis, the 10-year progression-free survival (PFS) rates of O_IDH_mut and A_IDH_mut are 62.5% ~ 71.7% and 30.4% ~ 38.3%, respectively^1^. The genomic and molecular alterations of IDH_mut oligodendroglioma and IDH_mut astrocytoma were well described by The Cancer Genome Atlas in 2015 and Ceccarelli et al. in 2017^2,3^ and introduced in neuropathological diagnosis. As a result, the world health organization (WHO) classification was revised, and these alterations became important biomarkers for the diagnosis of diffuse gliomas^4–6^.

Prior to the era of genetics-integrated diagnosis, astrocytomas and oligodendrogliomas were often confused with mixed oligoastrocytomas, but confusion including interobserver variability has now been greatly reduced, with A and O_IDH_mut being well-defined brain tumors. O_IDH_mut characteristically carries *IDH* mutation and 1p/19q codeletion, with additional mutations of capicua transcriptional repressor (*CIC*) and/or far upstream element binding protein 1 (*FUBP1*), telomerase reverse transcriptase promoter (*TERTp*) mutation, and O6-methylguanine methyltransferase promoter (*MGMTp*) methylation. IDH-mutant astrocytomas usually show alpha-thalassemia/mental retardation syndrome X-linked (*ATRX)* and tumor protein 53 (*TP53)* mutations^5^. However, it is unknown how often these gene variants occur in O_IDH_mut and A_IDH_mut. *CIC, FUBP1,* and *ATRX* variants are relatively limited, as these tumor-suppressor gene alterations are scattered throughout exons and introns. Although genetics-integrated diagnostics have greatly improved pathological diagnostic accuracy and significantly reduced interobserver variability^2,3^, there are still cases of morphological and genetic ambiguities, and the existence of mixed oligoastrocytoma is questionable. In 2014, loss of *CIC* and *FUBP1* expression was reported as a potential indicator for shorter relapse times in oligodendroglial tumors^7^. Nevertheless, this conjecture may lose credibility if *CIC* and/or *FUBP1* mutations are ubiquitous in oligodendrogliomas. First, this study aimed to elucidate the exact incidence and mutation types of *CIC* and FUBP1. Second, we aimed to show whether mixed oligoastrocytoma does exist or not. Third, we investigated the prognostic factors of adult-type diffuse gliomas. Finally, we explored through a literature review why *IDH1* mutation is not the only driver of diffuse gliomas but is usually accompanied by mutations in tumor-suppressor genes and telomere maintenance mechanisms.

## Materials and methods

A total of 169 cases of O_IDH_mut (n = 74) involving 70 patients and A_IDH_mut (n = 95) involving 90 patients were obtained from the archives of the Seoul National University Hospital (SNUH) from July 2018 to September 2022. Primary IDH-mutant astrocytomas were 18% more common than primary oligodendrogliomas (59% vs. 41%) over the last 3 years at a single institute (SNUH) when recurrent cases were excluded. Next-generation sequencing (NGS) using a customized FiRST (“Friendly integrated Research-based Smart Trustworthy”) brain tumor-targeted gene panel (BTP) was performed in all cases. A list of genes included in the FiRST BTP is provided in Supplementary Table 1. Two pathologists (SI Kim and SH Park) reviewed the genetics-integrated diagnoses with NGS results. Genetics-integrated diagnoses were made according to the 5th edition of the WHO classifications of the CNS published in 2021^5^. When the genetic features of A_IDH_mut and O_IDH_mut coexisted, methylation profile-based classification using the Deutsches Krebsforschungszentrum (DKFZ) algorithm was performed using Infinium MethylationEPIC 850K BeadChip array platforms.

The epidemiologic features of the samples are summarized in Table 1. Among the 74 O_IDH_mut cases, CNS WHO grades 2 and 3 were seen in 28 (37.8%) and 46 (62.2%) cases, respectively. Among the recurrent O_IDH tumors, 7 (9.5%) were grade 2 and 22 (29.7%) were grade 3. The male-to-female ratio was 1:1.1, and the median age was 45 years (range 19–68 years). The most common site was the frontal lobe in 56.8%, followed by multiple lobes (24.3%), the temporal lobe (8.1%), the insula (4.1%), the parietal lobe (4.1%), and the cingulate cortex (2.7%).

**Table 1:** Epidemiological summary and immunohistochemical result of studied IDH-mutant oligodendrogliomas and astrocytomas.

Oligodendroglioma	CNS WHO grade 2 (n = 28, 37.8%)	CNS WHO grade 3 (n = 46, 62.2%)		Total (n = 74)
Median age (years) (range)	44.5 (19 ~ 66)	45.5 (23 ~ 68)		45 (19 ~ 68)
Male to female ratio	1:1	1:1.1		1:1.1
Frontal lobe	60.7% (17/28)	54.3% (25/46)		56.8% (42/74)
Multiple lobes	21.4% (6/28)	26.1% (12/46)		24.3% (18/74)
Temporal lobe	7.1% (2/28)	8.7% (4/46)		8.1% (6/74)
Insula	7.1% (2/28)	2.2% (1/46)		4.1% (3/74)
Parietal lobe	3.6% (1/28)	4.3% (2/46)		4.1% (3/74)
Cingulate	0	4.3% (2/46)		2.7% (2/74)
Primary: recur	3:1	1.1:1		1.6:1
Median Ki-67 (range)	6.3% (2.2 ~ 7.5)	22.55% (13.4 ~ 60.4)		
Median mitotic rate (range)	2/10 HPF (0 ~ 7)	8/10 HPF (1 ~ 54)		

Astrocytoma	CNS WHO grade 2 (n = 16, 16.8%)	CNS WHO grade 3 (n = 43, 45.3%)	CNS WHO grade 4 (n = 36, 37.9%)	Total (n = 95)
Median age (years) (range)	48 (20 ~ 69)	40 (21 ~ 68)	40 (14 ~ 75)	41 (14 ~ 75)
Male to female ratio	2.2:1	1.2:1	2:1	1.6:1
Frontal lobe	68.8% (11/16)	39.5% (17/43)	47.2% (17/36)	47.4% (45/95)
Multiple lobes	18.8% (3/16)	20.9% (9/43)	36.1% (13/36)	26.3% (25/95)
Temporal lobe	0	20.9% (9/43)	2.8% (1/36)	10.5% (10/95)
Parietal lobe	6.3% (1/16)	7.0% (3/43)	8.3% (3/36)	7.4% (7/95)
Insula	6.3% (1/16)	7.0% (3/43)	0	4.2% (4/95)
Occipital lobe	0	0	2.8% (1/36)	1.1% (1/95)
Paracentral lobule	0	2.3% (1/43)	0	1.1% (1/95)
Lateral ventricle	0	2.3% (1/43)	0	1.1% (1/95)
Cerebellum	0	0	2.8% (1/36)	1.1% (1/95)
Median Ki-67 index (range)	2.1% (0.2 ~ 4.8)	7.4% (2.0 ~ 57.2)	28.6% (5.6 ~ 90.2)	8.8% (1.2 ~ 90.2)
Median mitotic rate (range)	1/10 HPF (0 ~ 2)	6/10 HPF (3 ~ 77)	12/10 HPF (4 ~ 108)	41/10 HPF (0 ~ 108)

Of the 95 A-IDH cases, CNS WHO grades 2, 3 and 4 were assigned to 16 (16.8%), 43 (45.3%), and 36 (37.9%), respectively. The male-to-female ratio was 1.6:1, and the median age was 41 years (range 14–75 years). The most common site was the frontal lobe (47.4%), followed by multiple lobes (26.3%), the temporal lobe (10.5%), the parietal lobe (7.4%), the insula (4.2%), the occipital lobe (1.1%), the central lobule (1.1%), the lateral ventricle (1.1%), and the cerebellum (1.1%).

### Immunohistochemical (IHC) study

Tissue sections were stained with an anti-ATRX polyclonal antibody (1: 300 dilution, ATLAS Antibodies AB, Bromma, Sweden), anti-GFAP (6F2) monoclonal antibody (1: 200 dilution, DAKO, Glostrup, Denmark), anti-IDH1 R132H (H09) monoclonal antibody (1:300 dilution, Dianova, Hamburg, Germany), anti-Ki67 (MIB-1) antibody (1:100 dilution, DAKO), anti-H3K27 M polyclonal antibody (1:700 Millipore, Temecula, USA), anti-p53 monoclonal antibody, DO-7 code M7001 (1:100 dilution, DAKO), anti-PHH3 antibody (1:100 dilution, Cell Marque, Rocklin, CA, USA) and anti-vimentin antibody (1:500, DAKO) (Supplementary Table 2). IHC staining was performed using a standard avidin–biotin-peroxidase method with a BenchMark ULTRA system (Roche Diagnostics, Indianapolis, US). The positive control was known positive tissue, and entrapped positive cells were used. For the negative control, the primary antibody was omitted.

*ATRX* gene mutations were scattered throughout exons and introns, making some sites difficult to capture using NGS. Since the initial version of our customized brain tumor panel could not detect full variants of *ATRX* mutations, we relied on ATRX IHC, but upgrade version of BTP panel could detect all the mutations.

### DNA extraction and NGS with customized brain tumor-targeted combined DNA/RNA gene panels

Representative areas of tumors with at least 90% tumor cell purity in hematoxylin and eosin-stained formalin-fixed paraffin-embedded (FFPE) sections were outlined for macrodissection. DNA was extracted according to the manufacturer's instructions from serial sections of the macrodissected tumor tissue using Maxwell® RSC DNA FFPE Kit (AS1450; Promega, USA) with a tumor-targeted gene panel (FiRST brain tumor panel and FiRST pancancer panel), as customized and verified by the Department of Pathology of Seoul National University Hospital (SNUH), and used with Hi-Output NextSeq550Dx. Panels 2 and 3 contained 172 and 207 genes, respectively, and 20 and 52 fusion genes, respectively. Sequencing data were analyzed using the SNUH FiRST Brain Tumor Panel Analysis pipeline. First, we performed quality control on the FASTQ file and analyzed only data that passed the criteria. Paired-end alignment to the hg19 reference genome was performed using BWA-men and GATK Best Practice^8^. After finishing the alignment step, an “analysis-ready BAM” was produced, and a second quality control was performed to determine if further variant calling was appropriate. Single-nucleotide variation (SNV), insertion and deletion (InDel), copy number variation (CNV), and translocation were analyzed using at least two analysis tools, including in-house and open-source software. The open-source tools used were GATK UnifiedGenotyper, SNVer and LoFreq for SNV/InDel detection; Delly and Manta for translocation discovery; THetA2 for purity estimation; and CNVKit for CNV calling, as previously described by Park et al*.*^9^. SnpEff was used to annotate the variants detected in various databases, including RefSeq, COSMIC, dbSNP, ClinVar, and gnomAD. Germline variants were then filtered using the population frequency of these databases (> 1% population frequency). Finally, the multidisciplinary molecular tumor board confirmed the variants through a comprehensive review. For RNA sequencing, tumor RNA was extracted from a paraffin block (tumor fraction: > 90%) using Maxwell® RSC RNA FFPE Kit (AS1440; Promega). The library was generated using SureSelectXT RNA Direct Kit (Agilent, Santa Clara, CA, USA) and sequenced using an Illumina NovaSeq 6000 at Macrogen (Seoul, Republic of Korea). Raw sequencing reads were analyzed using three algorithms, namely, DIFFUSE, Fusion catcher, and Arriba (https://github.com/suhrig/arriba/), to detect gene fusions, and the results were compared. FASTQ files were briefly aligned using the STAR aligner on the hg19 reference genome for Arriba analysis. A chimeric alignment file and read-through alignment file were produced, and fusion candidates were generated with a set of filters that detected artifacts based on various characteristic features.

### *MGMTp* methylation based on methylation-specific polymerase chain reaction (MSP) analysis

The MSP technique was used to analyze *MGMTp* methylation. Prepared DNA was modified via sodium bisulfite treatment using EZ DNA Methylation-Gold Kit (D5005; Zymo Research, Orange, CA, USA). The primer sequences used for *MGMTp* were as follows: methylated forward, 5′-TTT CGA CGT TCG TAG GTT TTC GC-3′; methylated reverse, 5′-GCA CTC TTC CGA AAA CGA AAC G-3′; unmethylated forward, 5′-TTT GTG TTT TGA TGT TTG TAG GTT TTT GT-3′; unmethylated reverse, 5′-AAC TCC ACA CTC TTC CAA AAA CAA AAC A-3′. The annealing temperature was set to 64 °C. The obtained polymerase chain reaction products were electrophoresed on 2% agarose gels and visualized under UV illumination after staining with ethidium bromide.

### Genomic alteration visualization

Clinical information, mutations, and CNVs were summarized using Oncoprint data, which were generated using the R package “ComplexHeatmap” (version 2.7.6.1002)^10^.

### Methylation array analysis and t-SNE clustering with the Illumina 850 K microarray

DNA methylation array analysis was performed for three ambiguous cases of O_IDH_mut using an Infinium MethylationEpic 850 K BeadChip array platform (Illumina, USA). DNA methylation data analysis was performed using R software (R 4.1.3, https://www.r-project.org/). Our samples were analyzed using the methylation classifier of DKFZ with reference to a diffuse glioma cohort of 551 samples from 10 subclasses^11^. A preprocessing procedure was conducted for raw methylation intensity signals using the R package “methylationArrayAnalysis” (version 1.14.0)^12^. We utilized the “combinedArray” command to merge the different platforms (450 K, EPIC). After filtering and normalization, 365,201 probes remained for subsequent analysis. The 20,000 most variably methylated probes were selected based on the standard deviation to perform unsupervised nonlinear dimension reduction. The resulting distance matrix was used as the input for *t*-distributed stochastic neighbor embedding analysis (t-SNE; Rtsne package version 0.15). The nondefault parameters used were distance = TRUE, perplexity = 30, θ = 0, and max_iter = 2000. A t-SNE plot was generated using the “ggplot2” package (version 3.3.3) for effective visualization. IDAT files were uploaded to versions v11b4 and v12.5 of the online CNS tumor methylation classifier (https://www.molecularneuropathology.org) for classification.

### Survival analysis

The median overall survival (OS) and progression-free survival (PFS) were obtained using the R package “survminer” (version 0.4.9). Survival analysis was performed in 70 patients with O_IDH_mut and 87 patients with A_IDH_mut. As most patients with O_IDH_mut were alive at the time of analysis during 1 to 244-months (median 34.7 months) follow-up period, overall survival (OS) could not be obtained.

### Ethics approval and consent to participate

The institutional review board (IRB) of Seoul National University Hospital (SNUH) approved this study (IRB No: 1906-020-1037) and has therefore been performed under the ethical standards set out in the 1964 Declaration of Helsinki and its subsequent amendments. As this study is a retrospective review of anonymized electronic medical records, pathology, and NGS data utilizing a brain tumor-specific somatic gene panel, IRB of SNUH (IRB No: 1906-020-1037) waived the informed consent for this study under the Korean Bioethics and Safety Act.

## Results

### Molecular study of oligodendroglioma, IDH-mutant and 1p/19q-codeleted

All O_IDH_mut samples (n = 74) showed ATRX expression and were positive for IDH1 (H09) by immunohistochemistry. Vimentin was mostly negative in tumor cells, but variable vimentin-positive glial cells were found in the margin of the tumor, the gray matter with infiltrating tumor cells, and some minigemistocytes.

NGS revealed *IDH* and *TERTp* mutations and 1p/19q whole-arm deletion in all O_IDH_mut samples. *IDH1* and *IDH2* mutations were found in 91.9% (68/74) and 8.2% (6/74) of patients, respectively (Table 2). C228T and C250T mutations in the *TERT* promoter were detected in 67.6% (50/74) and 32.4% (24/74), respectively, of patients. *CIC, FUBP1*, and both *CIC* and *FUBP1* mutations were found in 75.7%, 45.9%, and 30.0% of patients, respectively. The numbers of *CIC* and *FUBP1* variants were 70 and 36, respectively.

**Table 2:** Genomic profile in oligodendrogliomas in this study.

Genes	CNS WHO grade 2 (n = 28)		CNS WHO grade 3 (n = 46)		Total (n = 74)
	No of variant				
	Known variants	Novel variants	Known variants	Novel variants	Total
IDH mutation and 1p/19q co-deletion					100% (74/74)
*IDH1*					
R132H	92.9% (26/28)		91.3% (42/46)		91.9% (68/74)
*IDH2*					
R172K	3.6% (1/28)		8.7% (4/46)		6.8% (5/74)
R172M	3.6% (1/28)		0		1.4% (1/74)
*CIC*					
Missense	7.1% (2/28)	7.1% (2/28)	15.2% (7/46)	10.9% (5/46)	21.6% (16/74)
Frameshift	21.4% (6/28)	0	21.7% (10/46)	2.2% (1/46)	23.0% (17/74)
Multi-hit	25.0% (7/28)	3.6% (1/28)	6.5% (3/46)	8.7% (4/46)	20.3% (15/74)
Nonsense (Stop gain)	3.6% (1/28)	0	8.7% (4/46)	0	6.8% (5/74)
Intronic	3.6% (1/28)	0	4.3% (2/46)	0	4.1% (3/74)
Subtotal	23.0% (17/74)	4.1% (3/74)	35.1% (26/74)	13.5% (10/74)	75.7% (56/74)
*FUBP1*					
Nonsense (Stop gain)	17.9% (5/28)	0	13.0% (6/46)	0	14.9% (11/74)
Frameshift	7.1% (2/28)	3.6% (1/28)	8.7% (4/46)	2.2% (1/46)	10.8% (8/74)
Intronic	14.3% (4/28)	3.6% (1/28)	4.3% (2/46)	0	9.5% (7/74)
Multi-hit	3.6% (1/28)	0	8.7% (4/46)	0	6.8% (5/74)
Missense	3.6% (1/28)	0	0	2.2% (1/46)	2.7% (2/74)
Subtotal	17.6% (13/74)	2.7% (2/74)	23.0% (17/74)	2.7% (2/74)	45.9% (34/74)
Either *CIC* and *FUBP1*					93.2% (69/74)
Both *CIC* and *FUBP1*	13.5% (10/74)		16.2% (12/74)		30.0% (22/74)
*TERT* promoter					100% (74/74)
C228T	71.4% (20/28)		65.2% (30/46)		67.6% (50/74)
C250T	28.6% (8/28)		34.8% (16/46)		32.4% (24/74)
*MGMT promoter* methylated	100% (28/28)		93.5% (43/46)		95.9% (71/74)
*TP53*			4.3% (2/46)		2.7% (2/74)

*AA change* amino acid codon change, *CDS* coding sequence change, *VAF* variant allele frequency.

The *CIC* gene variants found comprised missense (21.6%), frameshift (23.0%), multihit (20.3%), nonsense (6.8%), and intronic (splicing site) (4.1%) mutations (Table 2). The FUBP1 gene exhibited 14.9% nonsense (stop gained), 10.8% frameshift, 9.5% intronic (splicing), 6.8% multihit, and 2.7% missense mutations. Among these variants, 22.9% (16/70 variants) of *CIC* and 11.1% (4/36 variants) of *FUBP1* were novel, which were found in 19 O_IDH_mut cases (5 WHO grade 2, 14 WHO grade 3). Sixteen and four novel variants of *CIC* and *FUBP1* are listed in Supplementary Table 3.

A total of 13 (86.7%) O_IDH_mut cases with novel *CIC* variants also showed additional pathogenic gene mutations, such as *FUBP1* (23.1%), *KRAS* (33.1%), *NOTCH1* (23.1%), *TP53* (15.4%), *PIK3CA* (7.7%), *SETD2* (7.7%), *BRAF* amplification (7.7%), and *PTEN* loss (8.3%), but two cases had no additional pathogenic variant. Among the 4 O_IDH_mut with *FUBP1* novel variants, one involved a pathogenic mutation in *KIT*, and the other one involved pathogenic *NOTCH1* mutation. The remaining two had no additional pathogenic gene mutations. Two cases of O_IDH_mut also exhibited *TP53* pathogenic mutations (3.4%) in addition to 1p/19q-codeletion.

**Figure 1:**
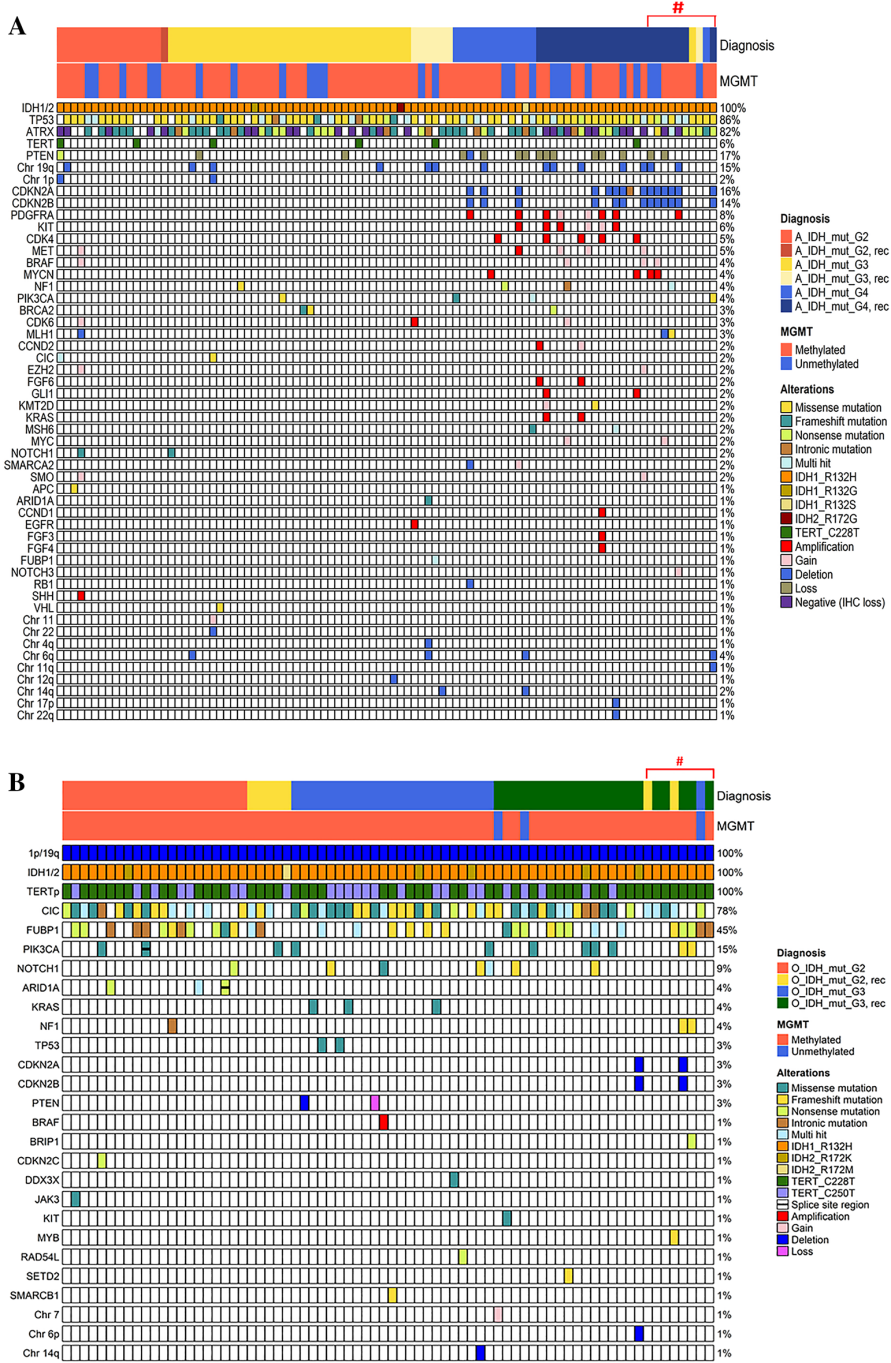
(**A**) The oncomap of IDH-mutant astrocytomas (n = 95) and (**B**) oligodendrogliomas (n = 74) of this study. The NGS study was performed twice on sequentially removed 18 tumors from 5 patients with A_IDH_mut and 4 patients with O_IDH_mut (#).

The rare mutations found in O_IDH_mut were *ARID1A, CDKN2C, JAK3, MYB, NF1, NOTCH1* and *PIK3CA* in CNS WHO grade 2 O_IDH_mut and *BRIP1, DDX3X, KIT, KRAS, NF1, NOTCH1, PIK3CA, RAD54L, SETD2*, and *SMARCB1* in CNS WHO grade 3 O_IDH_mut. This genomic landscape of O_IDH_mut is illustrated in Fig. 1A.

### Molecular study of astrocytoma, IDH-mutant

In 95 cases of A_IDH_mut, 86.3% carried a *TP53* mutation, 82.1% an *ATRX* mutation, and 6.3% a *TERTp* mutation (Table 3). The *IDH1/2* variants were R132H (96.8%), R132S (1.1%), R132G (1.1%) and *IDH2* R172G (1.1%). The variant types of *TP53* were missense in 58.9%, multihit in 16.8%, nonsense in 5.3%, frameshift in 3.2%, and intronic in 2.1% of cases.

**Table 3:** Genomic alterations found in astrocytoma, IDH-mutant in this study.

No of variants Genes	CNS WHO grade 2 (n = 16)	CNS WHO grade 3 (n = 43)	CNS WHO grade 4 (n = 36)	Total (n = 95)
*IDH1*				100% (95/95)
R132H (CGT > CAT)	100% (16/16)	95.3% (41/43)	97.2% (35/36)	96.8% (92/95)
R132S (CGT > AGT)			2.8% (1/36)	1.1% (1/95)
R132G (CGT > GGT)		2.3% (1/43)		1.1% (1/95)
*IDH2*				
R172G (AGG > GGG)	0	2.3% (1/43)	0	1.1% (1/95)
*TP53*	12.6% (12/95)	38.9% (37/95)	34.7% (33/36)	86.3% (82/95)
Missense	62.5% (10/16)	55.8% (24/43)	61.1% (22/36)	58.9% (56/95)
Multi-hit	12.5% (2/16)	14.0% (6/43)	22.2% (8/36)	16.8% (16/95)
Nonsense (Stop gained)	0	4.7% (2/43)	8.3% (3/36)	5.3% (5/95)
Frameshift	0	7.0% (3/43)	0	3.2% (3/95)
Intronic	0	4.7% (2/43)	0	2.1% (2/95)
*ATRX*#	11.6% (11/95)	31.6% (36/95)	32.6% (31/95)	82.1% (78/95)
Frameshift	31.3% (5/16)	30.2% (13/43)	25.0% (9/36)	28.4% (27/95)
Negative (IHC loss)	31.3% (5/16)	20.9% (9/43)	27.8% (10/36)	25.3% (24/95)
Nonsense (Stop gained)	0	23.3% (10/43)	16.7% (6/36)	16.8% (16/95)
Intronic	0	9.3% (4/43)	8.3% (3/36)	7.4% (7/95)
Multi-hit	6.3% (1/16)	0	5.6% (2/36)	3.2% (3/95)
Missense	0	0	2.8% (1/36)	1.1% (1/95)
*TERT* promoter				6.3% (5/95)
C228T	10.0% (2/16)	7.0% (3/43)	2.8% (1/36)	6.3% (6/95)
C250T	0	0	0	0
19q deletion only	6.3% (1/16)	9.3% (4/43)	22.2% (8/36)	13.7% (13/95)
1p deletion only	6.3% (1/16)	0	0	1.1% (1/95)
1p/19q-codeletion	0	2.3% (1/43)	0	1.1% (1/95)
*CDKN2A/2B*				
Homozygous deletion	0	0	38.9% (14/36)	22.6% (14/95)
*MYCN*				
Amplification	0	0	11.1% (4/36)	6.5% (4/95)
*PTEN*				24.2% (15/95)
Hemizygous deletion	0	4.7% (2/43)	33.3% (12/36)	22.6% (14/95)
Homozygous deletion	0	0	2.8% (1/36)	1.6% (1/95)
*PDGFRA*				8.4% (8/95)
Gain	0	0	5.6% (2/36)	2.1% (2/95)
Amplification	0	0	16.7% (6/36)	6.3% (6/95)
*CDK4 *amplification	0	0	13.9% (5/36)	5.3% (5/95)
*MET*				5.3% (5/95)
Gain	6.3% (1/16)		8.3% (3/36)	4.2% (4/95)
Amplification			2.8% (1/36)	1.1% (1/95)
*GLI1* amplification	0	0	5.6% (2/36)	2.1% (2/95)
*MGMT* promoter				
Methylated	68.8% (11/16)	79.1% (34/43)	69.4% (25/36)	73.7% (70/95)
Unmethylated	31.3% (5/16)	20.9% (9/43)	30.6% (11/36)	26.3% (25/95)

*IHC* immunohistochemistry.

^#^No novel mutation was found.

The *ATRX* mutation found by NGS and ATRX loss by IHC showed a 100% correlation in our cases. The *ATRX* mutation was found to be a frameshift in 28.4%, ATRX loss through IHC in 25.3%, nonsense (stop gained) in 16.8%, intronic (splicing) in 7.4%, multihit in 3.2% and deletion in 1.1% of cases.

The other CNVs found in A_IDH_mut are listed in Table 3. *PDGFRA* gain or amplification was detected in 8.4%, *CDK4* amplification in 5.3%, *MET* gain or amplification in 5.3%, *MYCN* amplification in 4.2% and *GLI1* amplification in 2.1% of cases. Chromosome 19q deletion, 1p deletion, and 1p/19q codeletion were found in 13.7%, 1.1% and 1.1% of cases, respectively.

Among the 17 cases of A_IDH_mut without *ATRX* mutation, *TERTp* mutation was observed in 29.4% (5 cases), *TP53* mutation in 76.5% (13 cases), and CNV of genes or chromosomes in 52.9% (9 cases) (Table 4). The amplified genes included *CDK4* (n = 3), *MYCN* (n = 2), *PDGFRA* (n = 1), and *GLI* (n = 1). Chromosomal aberrations included 7 gain, 11 gain, 19 deletion, 22 deletion, and 1p/19 codeletion. As expected, the higher the grade was, the higher the occurrence of CNVs was (chi-square test, p < 0.00001) (Supplementary Table 4). This genomic landscape of A_IDH_mut is illustrated in Fig. 1B.

**Table 4:** Genetic profile of 11 cases of *ATRX*-intact astrocytoma, IDH-mutant.

Case	TERTp (n = 5)	TP53-mutation (%: VAF) (n = 13)	Copy number variation (n = 9)	Others (%: VAF)
1	C228T	p.Arg273Cys, c.817C > T (84.2%)	MYCN amplification (X21) GLI amplification (X21) CDK4 amplification (X21)	
2	C228T	p.Val197Gly, c.590 T > G (7.5%) p.Arg273Cys, c.817C > T (6.7%)	Chr 19 deletion (1 copy)	FUBP1 p.Asp83_Ser84delins Ala, c.248_250 + 1delACTG (13.5%)
3	C228T	p.Glu224*, c.670G > T (85.7%)	Chr 11 gain, Chr 22 deletion, 1p/19q co-deletion	
4	C228T	p.Tyr220Cys, c.659A > G (7.5%)	Absent	CIC p.His264Arg, c.791A > G (83%)
5	C228T	Absent	Absent	
6	Absent	p.Val274Ala, c.821 T > C (91.1%)	PDGFRA amp (X16), CDKN2A/2B homozygous deletion PTEN homozygous deletion RB1 homozygous deletion	
7	Absent	p.His179Tyr, c.535C > T (88.0%)	MYCN amp (X20) CDKN2A/2B homozygous deletion PTEN hemizygous deletion Chr 19 deletion	
8	Absent	p.Phe113Val, c.337 T > G (56.5%)	EGFR amplification CDK4 amplification	
9	Absent	p.Gly59fs, c.168_175dupAGACCCAG (32.0%)	PTEN hemizygous deletion	
10	Absent	p.Arg282Trp, c.844C > T (93.8%)	CDK4 amplification	
11	Absent	p.Arg248Trp, c.742C > T (29.6%)	Chr 7 gain	
12	Absent	p.His179Arg, c.536A > G (80.2%)	Absent	
13	Absent	p.His193Arg, c.578A > G (59.8%)	Absent	
14	Absent	p.Phe113Val, c.337 T > G (55.79%)	Absent	APC p.Ala1492Thr, c.4474G > A (10.33%)
15	Absent	Absent	Absent	NF1 p.Leu2324*, c.6971 T > A (25.93%)
16	Absent	Absent	Absent	
17	Absent	Absent	Absent	

*VAF* variant allele frequency.

### Comparison of the molecular profiles of sequentially developed tumors

When comparing the molecular profiles of the 5 pairs of A_IDH_mut and 4 pairs of O_IDH_mut, no significant difference was noted. However, in one case each of A_IDH_mut and O_IDH_mut, the homozygous deletion of *CDKN2A/2B* was disappeared in the recurrent tumors that was present in the previous tumors.

### Hypermutated astrocytoma, IDH-mutant

There were three hypermutated A_IDH_mut tumors; two MMR-deficient primary astrocytomas and one temozolomide (TMZ)-induced hypermutated recurrent astrocytoma (grade 4).

The first patient was a 28-year-old male who initially had grade 3 A_IDH_mut. He received local RT (50.4 + 10.8 Gy) after initial resection of the tumor and a second RT (45 Gy/25 Fx) at 3 and a half years later when the tumor relapsed as WHO grade 4 A_IDH-mut. He underwent a total of 3 reoperations. This patient was not treated with TMZ. This patient harbored an *MLH1* germline mutation (p.Arg687Trp, c.2059C > T), and the tumor exhibited high microsatellite instability (MSI-H), corresponding to Lynch syndrome-associated, MMR-deficient A_IDH_mut. Nevertheless, the patient developed no tumor other than the brain tumor.

The second patient was a 15-year-old boy with A-IDH-mut, WHO grade 4 with *MSH6* germline mutation (p.Arg1172fs, c.3514dupA). The tumor showed MSI-H. He underwent CCNU + TMZ (6 cycles). He has had no recurrent brain tumors or other extracranial tumors during the 40-month follow-up.

The third patient initially had grade 3 A_IDH_mut at the age of 32 years. After receiving concurrent chemoradiotherapy (CCRT) with TMZ for 2 years, the tumor recurred, and he underwent a second CCRT. After six months, the recurrent tumor was removed, and A_IDH_mut, grade 4, was diagnosed. NGS of the recurrent tumor revealed hypermutation. Because the patient did not carry a germline mutation in the MMR gene, the case was judged to be TMZ-induced hypermutation.

### Study of histologically and genetically confusing cases with DNA methylation profiling

**Figure 2:**
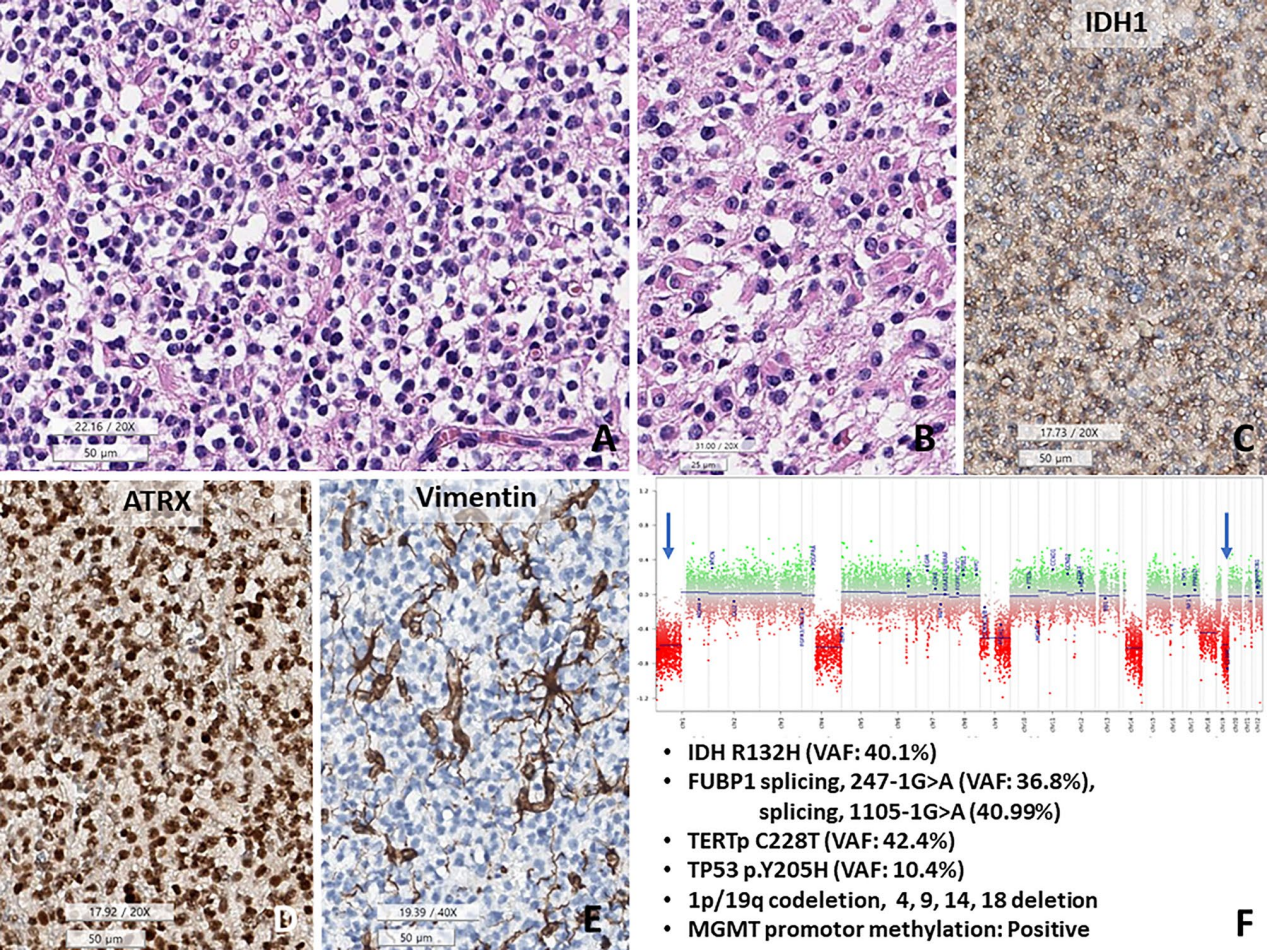
*TP53*-mutant oligodendroglioma, CNS WHO grade 3. (**A**) The tumor shows fried egg-appearing monotonous rounded cells with fine capillaries. (**B**) Some tumor cells have eosinophilic cytoplasm, consistent with minigemistocytic and gliofibrillary oligodendrocytes. (**C**–**E**) This tumor cells are positive for IDH1 and are retained expression of ATRX, but negative for vimentin. (**F**) Copy number variation plots obtained from methylation profiles show 1p/19q co-deletions and chromosomes 4, 9, 14, and 18 deletions (1 copy). In addition, *FUBP1* (splicing,247-1G>A, VAF 36.8%; splicing 1105-1G>A, 40.99%), *TERT* promotor mutation (C228T) and *MGMT* promoter methylation are present (**A**,**G** H&E, **C** ATRX IHC, **D** vimentin IHC, **E** CNV plot, under bar size A, **C**–**D** 50, B: 25 micrometer).

**Figure 3:**
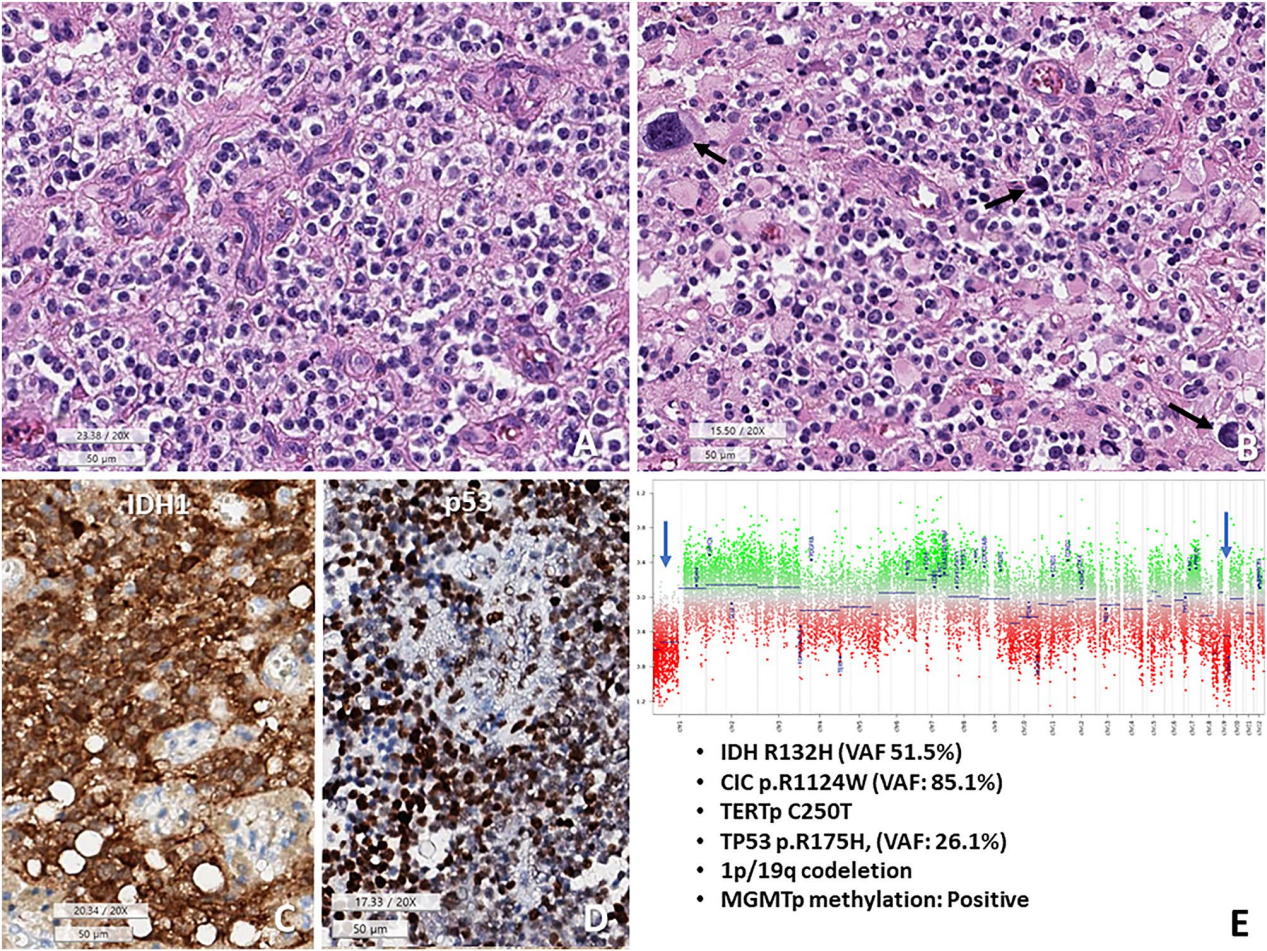
The second case of TP53 mutant oligodendroglioma, CNS WHO grade 3. (**A**) Tumor shows fried egg-appearing monotonous round cells with microvascular proliferation. (**B**) Some tumor cells have pleomorphic and hyperchromic nuclei (arrows) and abundant eosinophilic cytoplasm. This photo is a little bit out of focus because of nearby calcifications. (**C**,**D**) These tumor cells are positive for IDH1 (H09) and P53. (**E**) Copy number variation plots obtained from methylation profiles represent 1p/19q codeletion (one copy deletion). In addition, *CIC* (p.R1124W, VAF: 85.1%), *TERT* promoter (C250T), *TP53* (p.R175H, VAF: 26.1%) mutations and *MGMT* promoter methylation are present (**A**,**B** H&E, **C** IDH1 IHC, **D** p53 IHC, **E** CNV plot, under bar sizes **A**–**D** 50 micrometer).

**Figure 4:**
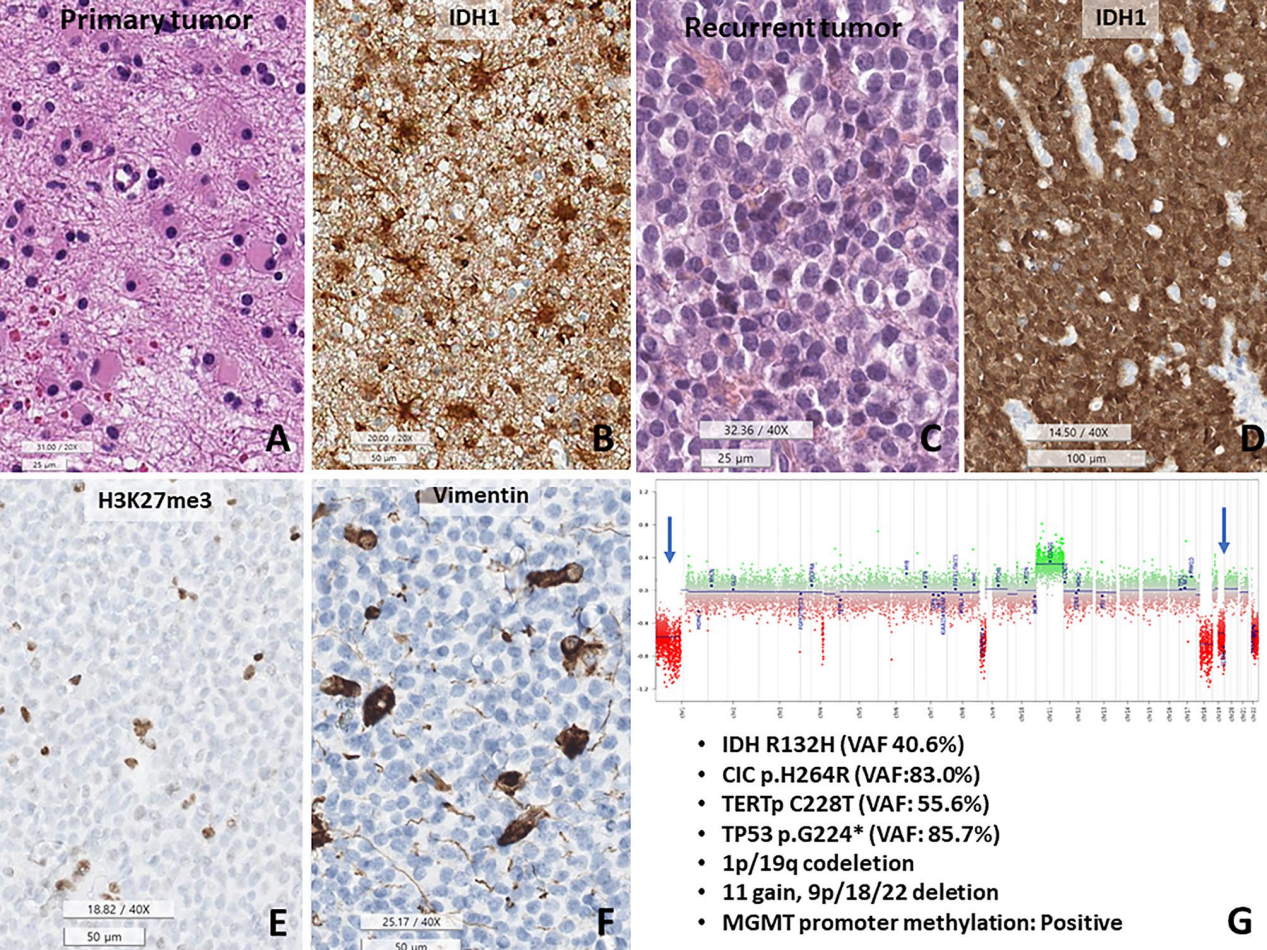
The 1p/19q-codeleted astrocytoma, IDH-mutant. This tumor recurred twice. (**A**,**B**) The initial tumor is a diffuse astrocytoma, IDH-mutant, consisting of hereditary astrocytes and IDH1-positive cells. (**C**,**D**) The second recurrent tumor shows morphological changes in the monotonous round shape with IDH1-positive round nuclei in the tumor cell nucleus. (**E**,**F**) Recurrent tumor cells are negative for H3K27me3 and vimentin, also reported in oligodendrocytes. (**G**) Copy number variation plots show 1p/19q-codeletion, chromosome 11 gain and 9p/18/22 deletion. This tumor also has *CIC* mutations (p.H264R, VAF: 83.0%), *TERT* promoter mutations (C228T), and *TP53* mutations (p.G224*, VAF: 85.7%), and *MGMT* promoter methylation (Under bar scale, **A**,**C**: 25, **B**,**E**,**F**: 50, **D**: 100 micrometer).

Although most O_IDH_mut cases were not difficult to diagnose, 2.7% (2/74) of O_IDH_mut and 1.1% (1/95) of A_IDH_mut cases in our study showed confounding with respect to genomic profiles and morphology (Figs. 2, 3, and 4). All three cases had *TP53* mutations, along with classic genomic signatures of O_IDH_mut; 1p/19q-codeletion, *TERTp* and *CIC,* mutations. The first two cases showed histopathological oligodendroglioma-like morphology with minigemistocytic or gliofibrillary oligodendrocytes. Remaining one patient had histopathologically CNS WHO grade 2 astrocytoma as the initial tumor but progressed to CNS WHO grade 3, with an ambiguous appearance with more rounded nuclei in the recurrent tumor (Fig. 4).

The first two cases (Cases #24 and #26) were matched with O_IDH_mut in two versions of methylation classifier (Case #24: v11b4: score 0.98853, v12.5: score 0.97911, Case #26: v11b4: score 0.79244, v12.5: score 0.78328) (Table 5). The methylation class of the remaining case was different according to the version of the DKFZ methylation classifier. Based on v11b4 DKFZ methylation classifier, it matched O_IDH_mut (score 0.75304) and did not match A_IDH_mut_HG (score 0.16219); however, with the recently updated v12.5 DKFZ algorithm, it matched diffuse glioma, *IDH* mutant (score: 0.92333), and A_IDH_mut_HG (score: 0.83527) (Table 5).

**Table 5:** The histologically and genetically confusing cases with DNA methylation profiling, which are TP53-mutant oligodendrogliomas and 1p/19q-deleted astrocytoma, IDH-mutant.

Case	O_IDH_mut_#24 (52y/male)	O_IDH_mut_#26 (52y/male)	A_IDH_mut_#62 (58y/male)
Pathol Dx	Oligodendroglioma	Oligodendroglioma	Astrocytoma, IDH-mutant
Site	Left frontal	Right frontal	Right temporooccipital
Histopathology	Oligodendroglioma-like, with ambiguous area	Oligodendroglioma-like, with ambiguous area	Initially astrocytic, ambiguous in recurrent tumor
Grade	3	3	3
IDH	R132H (40.1%)	R132H (51.5%)	R132H (40.6%)
TP53	p.Tyr205His, c.613 T > C (VAF: 10.4%), pathogenic	p.Arg175His, c.524G > A (VAF: 26.1%), pathogenic	p.Glu224*, c.670G > T (VAF: 85.7%), pathogenic
1p/19q	codeletion	codeletion	codeletion
TERTp	C228T (42.4%)	C250T (45.5%)	C228T (55.56%)
ATRX	No mutation	No mutation	No mutation
CIC		p.Arg1124Trp, c.3370C > T (85.1%): Likely pathogenic (ClinVar)	p.His264Arg, c.791A > G (83.%): Likely pathogenic
FUBP1	splicing, c.1105-1G > A (36.8%): Likely pathogenic, splicing c.1247-1G > A (31.3%): Likely pathogenic	X	X
Additional CNV	4, 9, 14, 18 deletion	X	11 gain, 9p/18/22 deletion
MGMTp	Methylated	Methylated	Methylated
MC v11b4	O_IDHm: 0.98853 (match)	O_IDHm: 0.79244 (match)	O_IDHm: 0.75304 (match)
	A_IDHm_HG: 0.00461 (no match)	A_IDHm_HG: 0.08600 (no match)	A_IDHm_HG: 0.16219 (no match)
MC v12.5	O_IDHm: 0.97911 (match)	O_IDHm: 0.78328 (match)	A_IDHm_HG: 0.83527 (match)
	A_IDHm_LG: 0.01109 (no match)	A_IDHm_HG: 0.15094 (no match)	O_IDHm: 0.06456 (no match)
Conclusion	O_IDHm	O_IDHm	A_IDHm_HG_1p/19q codeletion
Recurrence	No recur	No recur	2 times
Outcome	Survived in 38 months	DOD in 35 months	Survived in 84 months

*DOD* death of disease.

We performed a dimensional reduction procedure to visualize the results into t-SNE plot using a reference cohort of adult-type diffuse gliomas and these three cases whown in Table 5. Since the reference cohort data of the t-SNE plot was version v11b4 of DKFZ, one case of 1p/19q-codeleted A_IDH_mut clustered with O_IDH_mut, as mentioned above (Supplementary Fig. 1, t-SNE plot by v11b4); unfortunately, the t-SNE plot by v12.5 is not shown because we could not obtain the reference data by v12.5.

### Survival analysis

**Figure 5:**
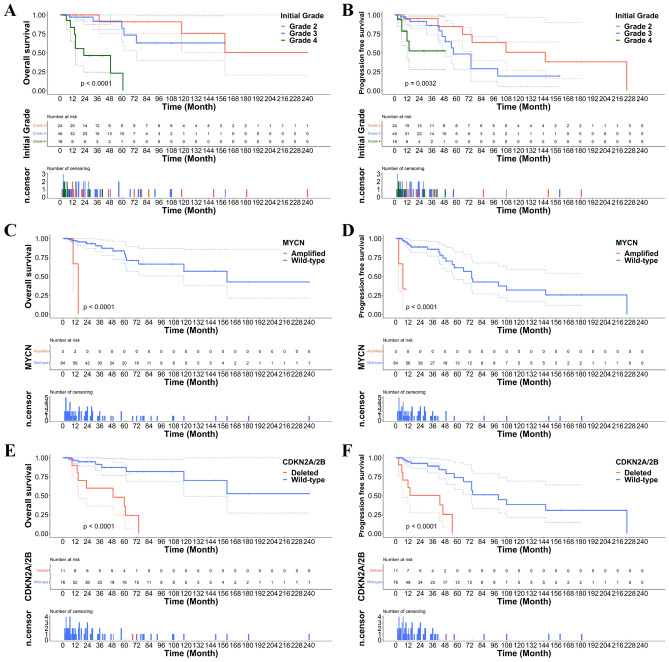
(**A**, **B**) Overall and progression-free survivals (OS and PFS) were significantly worse with increasing grade of A_IDH_mut. (**C**, **D**) OS and PFS were significantly worse in the patients with *MYCN*-amplified and (**E**, **F**) *CDKN2A/2B* homozygously deleted A_IDH_mut.

Kaplan–Meier survival analysis of the OS and PFS of patients with A_IDH_mut revealed significantly worse survival with increasing grade (Fig. 5A, B).

**Figure 6:**
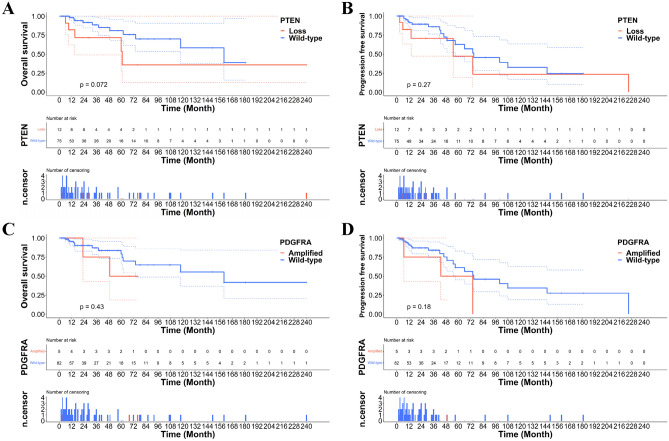
(**A**, **B**) *PTEN* deletion and (**C**, **D**) *PDGFRA* amplification do not affect the OS and PFS.

**Figure 7:**
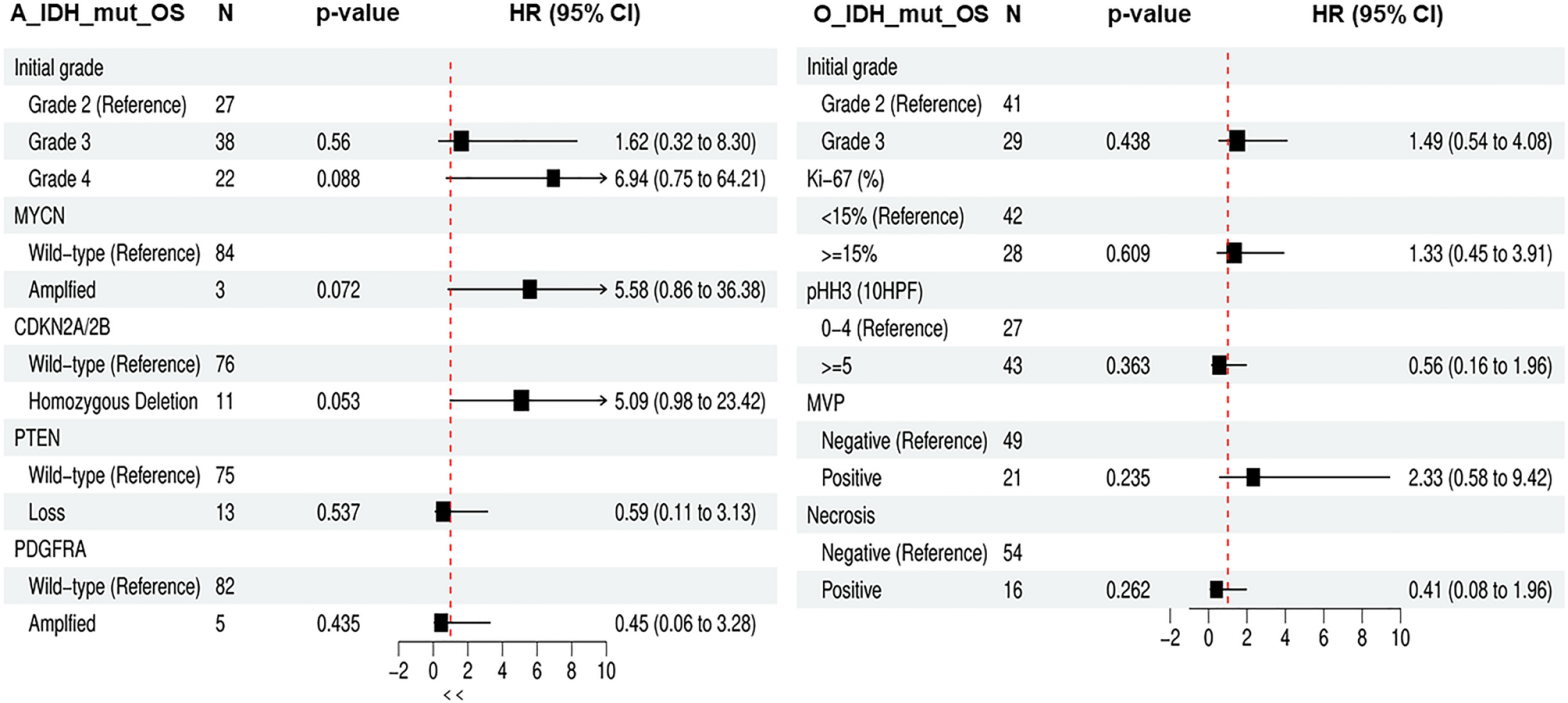
The hazard plot of various grade and genetic parameters in A_IDH_mut and O_IDH_mut. In A_IDH_mut, grade and *CDKN2A/2B* were statistically significant and *MYCN*-amplified tumor also were statistical significance, however, *PTEN* loss and *PDGFRA* amplification did not affect on the biological behavior of A_IDH_mut. In O_IDH_mut, the higher grade the higher HR, but it does not have statistical significance in this study (*OS* overall survival, *N* number of cases, *HR* hazard ratio, *MVP* microvascular proliferation).

Patients with A_IDH_mut with *CDKN2A*/*2B* homozygous deletion or *MYCN* amplification had significantly worse survival than those with A_IDH_mut without these two gene alterations (Fig. 5C–F). These results correspond to previous studies and cIMPACT-NOW updates 5 and 6^6,13–15^. However, *PDGFRA* amplification and *PTEN* loss did not affect patient survival in the A_IDH_mut group in our study (Fig. 6A–D). The HR and p-values of these parameters are provided in Fig. 7. No parameters for O_IDH_mut had significant hazard ratios.

## Discussion

A_IDH_mut and O_IDH_mut have characteristic histopathological, genetic and epigenetic profiles. Although it is well known that morphologically ambiguous cases exist, they can be diagnosed as A_IDH_mut or O_IDH_mut through a genetics-integrated diagnosis. This study aimed to determine whether mixed oligoastrocytoma occurs and whether “NOS” cases can be eliminated using genetics-integrated diagnostics and DKFZ methylation classifier.

### Function of oncometabolites produced by IDH mutation

The oncometabolite 2-hydroxyglutarate (2-HG), which is produced by *IDH1/2* mutations, is a competitive inhibitor of multiple alpha-ketoglutarate (a-KG)-dependent dioxygenases. 2-HG induces a wide range of histone demethylases at the promoter level and the ten eleven translocation (TET) family of dioxygenases of 5-methylcytosine (5mC) at the gene level^16^. The resulting chromatin compaction promotes the glioma CpG island hypermethylator phenotype (gCIMP), resulting in global silencing of tumor-suppressor genes (TSGs)^16,17^. Lu et al. reported that impaired histone lysine demethylation by accumulation of 2-HG caused by *IDH* mutation prevents cellular differentiation^18^. However, the exact mechanism of action of oncometabolites remains unknown. Unexpectedly, Filipski et al. and Habiba et al. found that *IDH1* R132H-mutant O_IDH_mut involves H3K27me3 protein loss in 96% (25/26) and 90% (36/40) of cases, respectively, but non-R132H IDH-mutant O_IDH_mut does not show H3K27me3 loss^19,20^. As mentioned above, loss of nuclear H3K27me3 is known to increase histone methylation by impairing demethylation of inhibitory histone markers induced by increased 2-HG levels in *IDH* mutant gliomas^18^. We believe that 2-HG may play a pivotal role in histone demethylation. CpG island DNA methylation in the nonpromoter region of IDH mutant cells, that is, DNA methylation of the CCCTC-binding factor (CTCF) binding site, results in loss of the insulated site, intercepts the topographically associated domain (TAD) boundary, and leads to an active enhancer in genes, such as *PDGFRA*^21^.

### Dysfunction of tumor-suppressor genes, *TP53* in astrocytoma, *CIC* and *FUBP1* in oligodendroglioma

*IDH* mutations are an early oncogenic molecular change in IDH-mutant gliomas. Mutation of *IDH* can cause oncogene (oncometabolite)-induced cellular senescence^22^. Constitutive activation of *RB1*, *TP53*, and *CDKN2A* is a well-known growth arrest signaling pathway that produces hypoproliferative senescent cells^23^. Senescent glioma cells adopt a different path to survive. Tumor cells need to block senescence-inducing TSGs, such as *TP53, RB1,* and *CDKN2A/2B*, so that can bypass senescence-induced tumor cell apoptosis. These molecular mechanisms may induce simultaneous *TP53* mutations in A_IDH_mut and *CIC* and/or *FUBP1* mutations in O_IDH_mut to obtain proliferative activity and to avoid cellular senescence.

*CIC* mutations are found in approximately 70% of oligodendrogliomas^24^, and we found them in 75.7% of our O_IDH_mut tumors. *CIC* is an important tumor suppressor that acts through transcriptional repression of target genes, including the polyoma enhancer activator 3 (PEA3) subfamily of E26 transformation-specific (ETS) transcription factors^24^. *CIC* is a transcriptional repressor that recruits histone deacetylations. *CIC* inactivation by mutations or deletion increases the level of histone acetylation, leading to transcription of EGFR/RAS/MAPK pathway components, promoting mitogen-independent tumor growth^24^.

*FUBP1* mutations are reported in approximately 15% of O_IDH_mut cases^25^, but they were found in 45.9% of O_IDH_mut cases in our series, including intronic mutations in 9.5%. Only four novel *FUBP1* mutations were identified in the present study (Table 2). FUBP1 was first described in 1994 as a single-stranded DNA-binding protein that binds to a noncoding, single-stranded far upstream element (FUSE) 2.5 kb upstream of the *MYC* promoter. *FUBP1* binds to the transcription factor IIH (TFIIH), activates the *MYC* oncogene, and directly represses p21 in normal hematopoiesis^26,27^. *FUBP1* is also a long-tail cancer driver that cooperates with other tumor-suppressor genes^28^. *FUBP1* is a posttranscriptional regulator of N6-methyladenosine (m^6^A) RNA methylation, translation, mRNA stability, and splicing. Its loss leads to global changes in RNA splicing and widespread expression of aberrant driver isoforms^28^. Therefore, somatic alteration of *FUBP1* (*FUBP1*^−/−^) contributes to neoplastic transformation via aberrant RNA splicing and m^6^A methylation^28^. *FUBP1* missense, nonsense, silent mutations, whole-gene deletions, frameshift deletions, and insertions have been observed in oligodendrogliomas^7^.

### Telomere lengthening by *ATRX* and *TERTp* mutations

Cancer involves rapidly proliferating cells that result in telomere shortening until the maximal number of cell divisions is reached (“Hayflick limit”)^22^. If replication proceeds, tumor cells gain chromosomal instability and eventually undergo apoptosis. For immortal growth, glioma cells exhibit telomere maintenance mechanisms (TMMs), which are *TERTp* mutation in O_IDH_mut and *IDH*-wildtype GBM and *ATRX* mutation in the majority of astrocytomas and histone-mutant pediatric-type high-grade gliomas^29^. *TERT* allows stabilization and elongation of telomeres. Altered length of telomeres (ALT) is another TMM that is induced by dysfunction of the *ATRX*/death-associated protein 6 (*DAXX*) complex^30^. However, *TERTp* mutation may be a second genetic event following oncogenic activation, such as *IDH* mutation in diffuse gliomas, *BRAF* V600E mutation in thyroid cancer, and *FGFR3* mutation in urothelial carcinoma^29,31,32^.

In our study, *TERTp* mutations were found in 100% of O_IDH_mut and 6.3% of A_IDH_mut cases, while *ATRX* mutations were present in 82.1% of A_IDH_mut cases but not in oligodendrogliomas. TERT has many functions, both canonical and noncanonical. The most significant canonical function of TERT is telomere lengthening, and the most important noncanonical functions of TERT are reduction of apoptosis, regulation of chromatin structure and gene expression^31^. Inhibition or inactivation of *CIC* by mutation is associated with *TERTp* mutation and increased *TERT* mRNA expression in O_IDH_mut^33^. *TERTp* and *ATRX* mutations are mutually exclusive, suggesting that they have equivalent TMMs^34^; however, questions of why O_IDH_mut involves *TERTp* mutation rather than *ATRX* mutation and most A_IDH_mut have *ATRX* mutations, not *TERTp* mutations, remain. In patients with A-IDH-mut, there was no difference in OS or PFS between the those with *ATRX* mutation and *TERTp* mutation (Supplementary Fig. 2A,B). *ATRX*- or *TERTp*-wildtype in patients with A_IDH_mut were associated with worse OS (p = 0.046) but did not affect PFS (Supplementary Fig. 2C–F).

### *MGMTp* methylation

DNA methylation patterns are generally stable and unique in differentiated cells; however, methylation profiles can be altered by extrinsic or intrinsic factors^35^. Both hypermethylation and hypomethylation play essential roles in long-term gene regulation and reactivation of oncogenes, TSG inhibition, deregulation of mRNA expression, mutagenesis, or alteration of functional chromosomal stability in cancers^36^. Genome-wide DNA methylation patterns can be used to subclassify brain tumors, which correlate with mutational status, DNA copy-number aberrations, and gene expression signatures^37,38^.

*MGMT* is a key DNA repair enzyme that removes mutagenic methyl groups from the P6 position of guanine by transferring it to the cysteine acceptor site of the protein itself^39^. *MGMTp* methylation is a better prognostic factor for gliomas^40^ and was eventually confirmed as a predictive marker for alkylating agents by European Organization for Research and Treatment (EORTC)^41^. *MGMTp* methylation belongs to the glioma CIMP family and was found in 95.9% of our O_IDH_mut series, and loss of nuclear H3K27me3 can be generated using IDH mutation-induced demethyltransferase blockade. According to Horbinski et al.’s study, the average beta values of O_IDH_mut of all 147 *MGMTp* CpG sites were significantly higher at 44.9%, and transcriptionally sensitive regions were 69% more methylated in O_IDH_mut. However, none were significantly more methylated in astrocytomas^42^. It is curious why *MGMTp* methylation appears to be less frequent (73.7%) in A_IDH_mut than in O_IDH_mut.

### Gene dosage effect in gliomas

**Figure 8:**
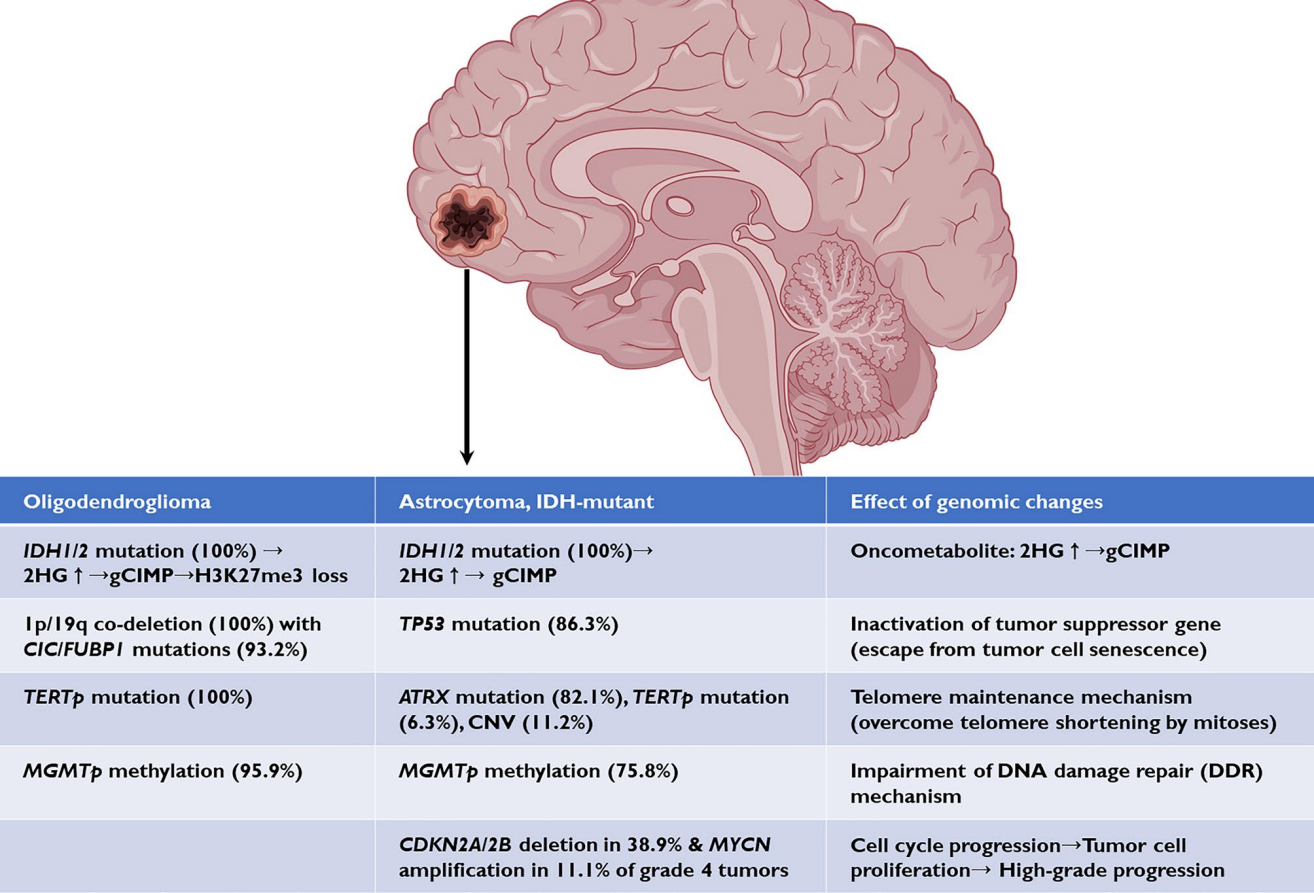
The summary of the genetic profiles and its effects in A_IDH_mut and O_IDH_mut.

In this study, higher grade A_IDH_mut showed significantly more CNVs than lower grade A_IDH_mut (Supplementary Table 3). A comprehensive genomic landscape revealed the gene dosage effect of gliomas, with more gene mutations resulting in more aggressive tumors^43^. A summary of the genomic features and possible effect of the molecular changes in O_IDH_mut and A_IDH_mut in our study is shown in Fig. 8.

## Conclusion

The combined oncometabolite 2-HG induced by IDH mutations, TSG blockade, and TMM are important drivers and diagnostic hallmark of IDH-mutant gliomas. Despite reliable histological or molecular characteristics of our IDH-mutant gliomas, there were doubts in three cases because of the mixed features of O_IDH_mut and A_IDH_mut. The methylation classifier of DKFZ (v.12.5) matched perfectly with either O_IDH_mut or A_IDH_mut, and no genetically or epigenetically ambiguous IDH-mutant adult-type diffuse gliomas were observed. These valuable features should be recognized in differential diagnosis and genotype-specific therapeutic strategy. Finally, patients with *MYCN*-amplified A_IDH_mut had the worst prognosis, strongly suggesting that *MYCN* amplification, in addition to *CDKN2A/2B* homozygous deletion, should be included in the genetic criteria for grade 4 A_IDH_mut.

## Supplementary Information

Supplementary Information.

## Data Availability

The datasets generated and/or analysed during the current study are available in the Gene Expression Omnibus (GEO) repository under accession number GSE222423 (https://www.ncbi.nlm.nih.gov/geo/query/acc.cgi?acc=GSE222423).

## Abbreviations

A_IDH_mut: Astrocytoma, IDH-mutant
BTP: Brain tumor-targeted gene panel
CNS: Central nervous system
WHO: World health organization
DKFZ: Deutsches Krebsforschungszentrum
FFPE: Formalin-fixed paraffin-embedded
GBM: Glioblastoma, IDH-wildtype
IHC: Immunohistochemical study, immunohistochemistry
NGS: Next-generation sequencing
NOS: Not otherwise specified
NEC: Not elsewhere classified
O_IDH_mut: Oligodendroglioma, IDH-mutant and 1p/19q-codeleted
OS: Overall survival
PFS: Progression-free survival
SNUH: Seoul National University Hospital
TMMs: Telomere maintenance mechanisms
