# Supplementary material for: Genomic profiles of IDH-mutant gliomas: MYCN-amplified IDH-mutant astrocytoma had the worst prognosis
Source: Sci Rep. 2023 Apr 25;13:6761. doi: 10.1038/s41598-023-32153-y (PMC10130138; doi:10.1038/s41598-023-32153-y)

Supplementary Table 1. List of the FIRST brain tumor panel established by the Department of Pathology, Seoul National University Hospital (FIRST means Friendly, Integrated, Research-based, Smart and Trustworthy).

| **DNA** | | | | | | | | | | **RNA** | | |
| --- | --- | --- | --- | --- | --- | --- | --- | --- | --- | --- | --- | --- |
| ACVR1 | C11ORF95 | CTNNB1 | FAT1 | HHIP | MAP2K2 | MYL1 | POLE | SFRP1 | TSC1 | ALK | HMGA2 | SS18 |
| ADAM29 | CBL | DAXX | FBXW7 | HIST1H3B | MAPK1 | NEGR1 | POLQ | SHH | TSC2 | AXL | MET | STAT6 |
| ADGRB3 | CCND1 | DDX3X | FGF3 | HIST1H3C | MAPK3 | NF1 | PPM1D | SMAD2 | UNC5D | BCOR | MN1 | TAF15 |
| ADGRG4 | CCND2 | DICER1 | FGF4 | HRAS | MDM2 | NF2 | PPP2R2A | SMAD4 | USP8 | BRAF | MYB | TFE3 |
| AIP | CCND3 | DIDO1 | FGF6 | IDH1 | MDM4 | NOTCH1 | PRDM6 | SMARCA2 | VHL | C11ORF95 | NRG1 | TGFBR3 |
| AKAP6 | CD300C | DKK2 | FGFR1 | IDH2 | MED12 | NOTCH2 | PRKAR1A | SMARCA4 | WIF1 | CIC | NTRK1 | TTYH1 |
| AKT1 | CD79A | DPYD | FGFR2 | IMPG2 | MEN1 | NOTCH3 | PRKCA | SMARCB1 | YAP1 | DDIT3 | NTRK2 | WHSC1 |
| ALK | CDH1 | EGFR | FGFR3 | JAK1 | MET | NPR3 | PTCH1 | SMARCE1 | ZIC1 | DDX31 | NTRK3 | YAP1 |
| APC | CDK12 | EMX2 | FGFR4 | JUN | MLH1 | NRAS | PTCH2 | SMO | ZMYM3 | EGFR | NUTM1 |  |
| ARID1A | CDK4 | EOMES | FLG | KBTBD4 | MLH3 | NRL | PTEN | SSTR2 |  | ERBB4 | PCSK5 |  |
| ARID1B | CDK6 | EPHA7 | FUBP1 | KCNA1 | MN1 | NTRK1 | PTPN11 | STAG2 |  | ERG | PDGFRA |  |
| ARID2 | CDKN1A | ERBB2 | GABRA5 | KDM5C | MRE11A | NTRK2 | RAD51B | STAT3 |  | ETV1 | PDGFRB |  |
| ATM | CDKN1B | ERCC2 | GAD1 | KDM6A | MSH2 | NTRK3 | RAD51C | STAT6 |  | ETV4 | PIK3CA |  |
| ATOH1 | CDKN2A | ERG | GFI1 | KHDRBS2 | MSH3 | OTX2 | RAD51D | STK11 |  | EWSR1 | PKD1 |  |
| ATRX | CDKN2B | ETV6 | GFI1B | KIT | MSH4 | PALB2 | RAD54L | SUFU |  | FGFR1 | PPARG |  |
| BARD1 | CDKN2C | EWSR1 | GLI1 | KLF4 | MSH5 | PDGFRA | RB1 | SYNCRIP |  | FGFR2 | PRKCA |  |
| BCL3 | CHEK1 | EYA1 | GLI2 | KMT2C | MSH6 | PDGFRB | RBM24 | TBR1 |  | FGFR3 | PVT1 |  |
| BCOR | CHEK2 | EZH2 | GNAS | KMT2D | MTOR | PDZD2 | RELA | TCF4 |  | FOXO1 | RAF1 |  |
| BRAF | CIC | FAM175A | GPR101 | KRAS | MYB | PIK3CA | RET | TERT |  | FOXR2 | RELA |  |
| BRCA1 | CREBBP | FANCA | GSE1 | MAB21L2 | MYBL1 | PMS1 | RGPD3 | TNC |  | FUS | RET |  |
| BRCA2 | CSNK2B | FANCD2 | H2AFX | MACF1 | MYC | PMS2 | ROS1 | TP53 |  | GFI1 | ROS1 |  |
| BRIP1 | CTDNEP1 | FANCL | H3F3A | MAP2K1 | MYCN | POLD1 | SETD2 | TRAF7 |  | GFI1B | SLC44A1 |  |

Supplementary Table 2. The list of the primary antibodies used in this study.

| Antibody | Dilution | Antigen retrieval | Clone | Source |
| --- | --- | --- | --- | --- |
| ATRX | 1: 300 | Ventana CC1 at 100^o^C | Polyclonal | Atlas antibodies, AB, Bromma, Sweden |
| GFAP | 1: 200 | Ventana CC1 at 100^o^C | 6F2 (monoclonal) | DAKO, Glostrup, Denmark |
| IDH1 | 1: 100 | Ventana CC1 at 100^o^C | H09 (monoclonal | Dianova, Hamburg, Germany |
| Ki67 | 1: 100 | Ventana CC1 at 100^o^C | MIB-1 (monoclonal) | DAKO, Glostrup, Denmark |
| K27M | 1: 700 | Ventana CC1 at 100^o^C | Polyclonal | Millipore, Temecula, USA |
| P53 | 1:100 | Ventana CC1 at 100^o^C | DO7 (monoclonal) | DAKO, Glostrup, Denmark |
| pHH3 | 1:100 | Ventana CC1 at 100^o^C | Polyclonal | Cell Marque, Rocklin, USA |
| Vimentin | 1: 500 | Ventana CC1 at 100^o^C | V9 (monoclonal) | DAKO, Glostrup, Denmark |

Abbreviation: GFAP, glial fibrillary acidic protein; K27M, antibody for Histone Lys27Met; pHH3, phosphorylated Histone H3, RTU: ready to use

Supplementary Table 3. The lists of novel mutations of CIC and FUBP1 found in CNS WHO grade 2 and 3 O_IDH_mut.

| O_IDH_mut | **CNS WHO grade 2 (pt. n=3)** | **CNS WHO grade 3 (pt. n=11)** |
| --- | --- | --- |
| *CIC (*VAF %) | p.Arg2421Gly, c.7261C>G (15.07%)  p.Ser132fs, c.393-395delCTCinsTT (67.5%)  p.Thr1375Pro, c.4123A>C (14.91%) | p.His681Pro, c.2042A>C (13.53%)  p.Arg1124Trp, c.3370C>T (85.11%)  p.His681Pro, c.2042A>C (19.56%)  p.His681Pro, c.2042A>C (15.73%)  p.His681Pro, c.2042A>C (22.38%)  p.Thr2284Pro, c.6850A>C (11.68%)  p.Thr1375Pro, c.4123A>C (11%)  p.Phe1484del, c.4450_4452delTTC (62.33%)  p.Arg202Trp, c.604C>T (55.72%)  p.Lys1517del, c.4550_4552delAGA (64.29%)  p.Arg1515His, c.4544G>A (13.81%) |
| *FUBP1* (VAF %) | 3’UTR, c.*139_*140delTT(35.9%)  p.Arg37fs, c.111_112delAG (33.3%) | p.Ala603fs, c.1807delG (3.07%)  p.Pro10Leu, c.29C>T (7.3%) |

Pt.: patient

Supplementary Table 4. Relationship between ATRX mutation status and CNV in astrocytomas, IDH-mutant.

| A_IDH_mut | CNS WHO grade 2  (n=16) | CNS WHO grade 3  (n=43) | CNS WHO grade 4  (n=36) | Total (n=95) |
| --- | --- | --- | --- | --- |
| With CNV | 3/16 (18.8%) | 10/43 (23.3%) | 27/36 (75.0%) | 40/95 (42.1%) |
| Without CNV | 13/16 (81.2%) | 33/43 (76.7%) | 9/36 (25.0%) | 55/95 (57.9%) |
| *p-value*# |  |  |  | < 0.00001 |
| ATRX-wt |  |  |  |  |
| With CNV | 1/16 (6.3%) | 4/43 (9.3%) | 4/36 (11.1%) | 9/95 (9.5%) |
| Without CNV | 4/16 (25.0%) | 3/43 (7.0%) | 1/36 (2.8%) | 8/95 (8.4%) |
| ATRX-mut |  |  |  |  |
| With CNV | 2/16 (12.5%) | 6/43 (14.0%) | 23/36 (63.9%) | 31/95 (32.6%) |
| Without CNV | 9/16 (56.3%) | 30/43 (69.8%) | 8/36 (22.2%) | 47/95 (49.5%) |
| *p-value*# |  |  |  | 0.9973 |
| *p-value*## | 1 | 0.0399 | 1 |  |

# *P-value* was obtained by Chi-squared test (The result is significant at p < .05), ##: *P-value* was obtained by Fisher's exact test (The result is significant at p < .05),

**Supplementary Fig. 1**


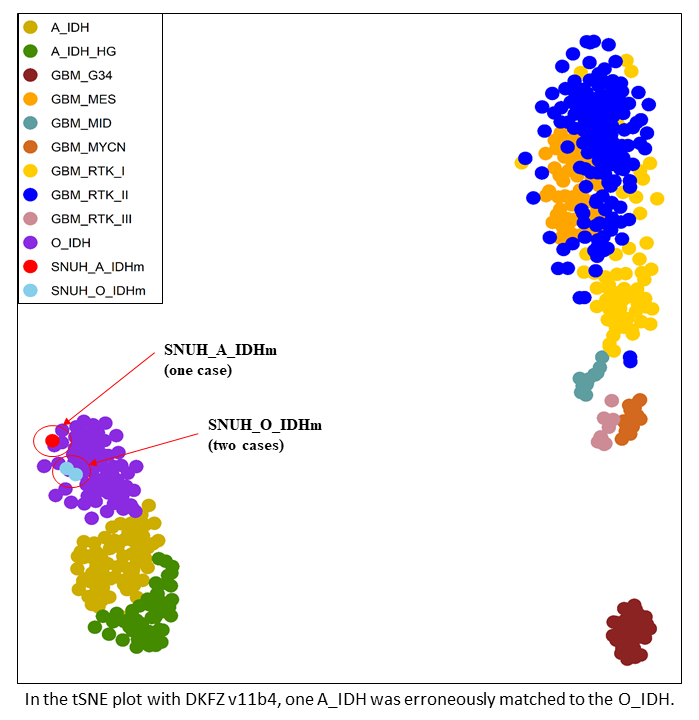


**Supplementary Figure 1**. TSNE plot of our 3 genetically confusiong cases by a dimensional reduction procedure using the reference cohort of adult-type diffuse gliomas. Since the reference cohort data of the t-SNE plot was version v11b4 of DKFZ, one case of 1p/19q-codeleted A_IDH was wrongly clustered to O_IDH.

**Supplementary Fig. 2.**

The effect of ATRX and/or TERT promoter mutation on OS and PFS in A_IDH-mut.


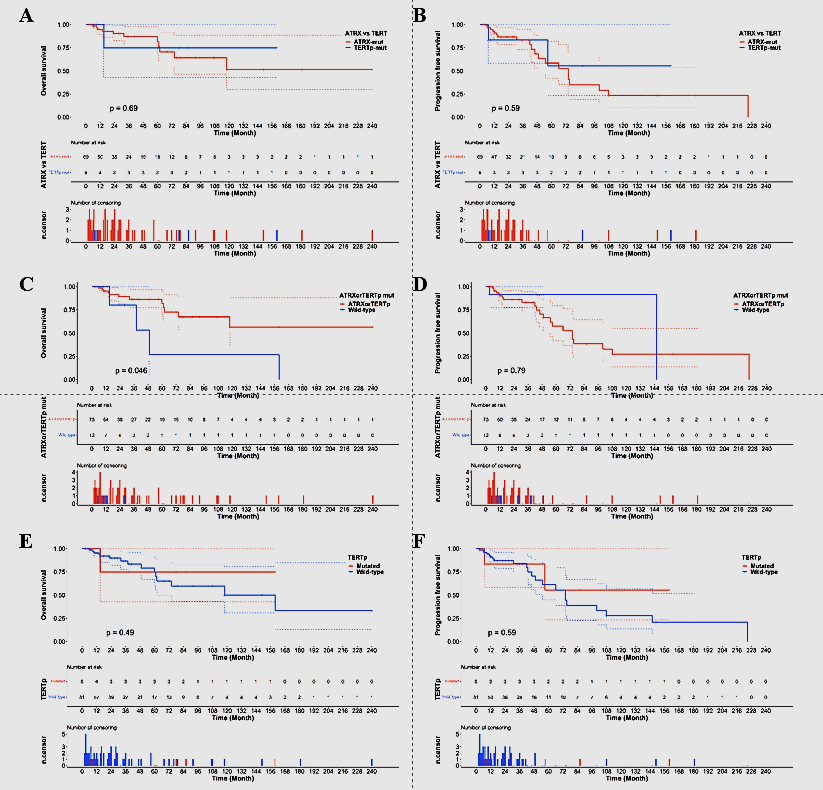

Supplement: Supplementary file 1 — Supplementary Information. [file 41598_2023_32153_MOESM1_ESM.docx]
